# A Multimodal Software Architecture for Serious Exergames and Its Use in Respiratory Rehabilitation

**DOI:** 10.3390/s23218870

**Published:** 2023-10-31

**Authors:** Claudinei Dias, Jhonatan Thallisson Cabral Nery, Marcelo da Silva Hounsell, André Bittencourt Leal

**Affiliations:** Department of Electrical Engineering, Santa Catarina State University, Joinville 89219-710, Brazil; jhonatanthallisson@gmail.com (J.T.C.N.); marcelo.hounsell@udesc.br (M.d.S.H.); andre.leal@udesc.br (A.B.L.)

**Keywords:** serious exergames, respiratory rehabilitation, architecture, devices

## Abstract

Serious Exergames (SEGs) have been little concerned with flexibility/equivalence, complementarity, and monitoring (functionalities of systems that deal with a wide variety of inputs). These functionalities are necessary for health SEGs due to the variety of treatments and measuring requirements. No known SEG architectures include these three functionalities altogether. In this paper, we present the 123-SGR software architecture for the creation of an SEG that is appropriate to the needs of professionals and patients in the area of rehabilitation. An existing SEG was adapted and therapy-related sensor devices (Pneumotachograph, Manovacuometer, Pressure Belt, and Oximeter) were built to help the patient interact with the SEG. The architecture allows the most varied input combinations, with and without fusion, and these combinations are possible for both conscious and unconscious signals. Health and Technology professionals have assessed the SEG and found that it had the functionalities of flexibility/equivalence, complementarity, and monitoring, and that these are really important and necessary functionalities. The 123-SGR architecture can be used as a blueprint for future SEG development.

## 1. Introduction

Serious games (SGs) are digital games with a specific purpose that extends beyond entertainment [1] and can be categorized into several types, such as games for learning, training, political/social issues, and health, among others. Exergames are games that employ some form of physical activity as interface control [2]. The engagement with exergames stimulates players to interact with the game challenges, thereby establishing a distinctive platform that seamlessly integrates hardware and software technologies into physiotherapy and enjoyment [3]. In this context, “digital technologies” refer to the software and hardware components that construct the gaming environment. “Physical exercise” denotes the physical efforts undertaken by players to interact with the game. “Fun” encapsulates the element of enjoyment and amusement derived from participating in the game. Exergames for respiratory rehabilitation are coming to smartphones and are incorporating new ways to catch respiratory signals, which emphasizes the need for better ways to accommodate a variety of signals in such applications; they can be effectively employed to simulate inspiratory flows through the utilization of incentive spirometry in a respiratory therapy context. Exergames enable individuals to engage in therapeutic exercises designed to promote and enhance respiratory function by encouraging users to perform inspiratory maneuvers using incentive spirometers within the gaming environment [4]. Serious Exergames (SEG) are exergames that have a purpose, beyond entertainment. Health is one of the relevant fields for the application of SEGs, resulting in a large number of games produced for the sector: a total of 1743 “Health Games” were developed in 23 countries by 2016 [5]. Some research studies have encouraged serious games played with devices (sensors and actuators), enhancing the gaming experience’s ubiquity, and have discussed the standard architecture, key technologies, and potential challenges [6].

One of the health aspects is rehabilitation, which aims to recover patients’ lost abilities. In recent decades, studies have emerged that promote rehabilitation through the use of SEGs as a driving force, among them are SGs for motor rehabilitation. Serious Exergames (SEG) help relieve symptoms resulting from stroke [7], Parkinson’s disease, and Amyotrophic Lateral Sclerosis, and in rehabilitating frail elderly patients [8], for instance. There are also SGs that assist in health-related issues such as diabetes [9] and obesity [10], and also SGs for respiratory rehabilitation, which relieves the symptoms of Cystic Fibrosis, Chronic Obstructive Pulmonary Disease [11], and Asthma, or supports the treatment of various respiratory disorders [12] (which include COVID-19-related long-term side effects). In addition, the physiological reactions promoted by exergames can help improve decision making and maintain a certain level of difficulty to make the activity more challenging and therefore more engaging and rewarding [13].

Among SEGs, there are those that are focused on rehabilitation (SGRs—Serious Games for Rehabilitation) and those focused on education, for instance. SEGs offer a compelling and valuable alternative for rehabilitation techniques, capitalizing on engaging, motivating, and immersive strategies to facilitate interventions. To further enhance the efficacy of such approaches, the integration of Multimodal Interaction Systems becomes paramount. These systems encompass the use of at least two distinct input and/or output modalities (e.g., from different sensors, e.g., the heart, brain, etc.), holding the potential to elevate the user experience and improve therapeutic outcomes. However, despite the promising capabilities of Multimodal Interaction Systems, certain critical functionalities in rehabilitation games remain underutilized in current SEG implementations [14], such as

- **Flexibility/Equivalence:** Without flexibility, the execution of an SEG for rehabilitation would be limited to a single controller device, restricting the therapy’s applicability. For instance, inspiratory flow or pressure could be triggered by some game action but they require different devices to be used, otherwise there is no such flexibility. To enhance this scenario, the architecture allows for multiple interaction modalities (forms of control for the SEG), providing therapists with diverse options for patient interaction [15]. For instance, consider a scenario where a physiotherapist is working with two patients requiring respiratory rehabilitation. Patient A faces no difficulties using conventional hand controllers, but the Manovacuometer Device proves suitable for their needs. In contrast, Patient B, with limited mobility, prefers an Extensor Belt Device for enhanced accessibility during therapy sessions. Flexibility enables therapists to cater to the unique needs of both patients by selecting the most suitable interaction modality for each case.
- **Complementarity (more complete information):** Through the combination of different sources of information about a phenomenon, one can reach the best understanding of it [14]. The acquisition of multimodal data often occurs in the medical context, as complementary information leads to better diagnosis and treatment. In addition, exploring complementary modalities simultaneously allows for better detail about the patient’s condition [16]. For example, when performing movements for physical rehabilitation, the patient can use compensatory movements to achieve the objectives of the exercises, masking the efficiency of the treatment. When playing a game, the patient is focused on the activity of the game and their attention to the correctness of the movement can be decreased, resulting in an increase in the number of incorrect moves or compensatory actions. Complementarity provides greater clarity of information about the patient’s performance during therapy, which can also cause incorrect patterns of therapeutic exercises to be detected and corrected [15].
- **Monitoring:** When using an SEG for rehabilitation, or for health promotion in general, where some kind of physical activity is required, it is possible that excessive effort may occur, or another factor could cause patient discomfort or even something more harmful that hinders their therapy. As an example, during prolonged exercise it is possible to experience hyperventilation, which is a condition in which one begins to breathe very fast. Severe hyperventilation can lead to loss of consciousness or result in underlying problems [16]. Another example is when instead of breathing quickly, one unconsciously stops breathing during exercise. This can produce a sharp increase in blood pressure, followed by a sudden drop, and cause dizziness or fainting [17]. Also, when exercise compensation occurs (when the patient does not perform an exercise correctly), it leads to injuries. This should then be highlighted if it reaches a certain threshold of repetitions. To avoid unwanted consequences, it is important to monitor possible side effects during the use of SEGs and create mechanisms to reverse some situations by [12] (a) warning about these cases; (b) slowing down the pace of stimuli; or even (c) stopping therapy. Monitoring physiological parameters, such as oxygen saturation and heart rate, can help ensure patient safety during the use of an SEG for rehabilitation [11].

Within the domain of Serious Exergames (SEG) for health, the significance of these three essential functionalities becomes evident. To address and enhance these critical aspects, we have thoroughly explored the integration of Multimodal Interaction Systems. Notably, one of the key advantages of multimodal SEGs lies in their emphasis on safety and the potential for long-term benefits. By incorporating multiple modalities, these systems create a safer and more immersive gaming experience, fostering increased user engagement. Moreover, the utilization of various modalities allows for a comprehensive and personalized approach to rehabilitation, ultimately contributing to improved long-term therapeutic outcomes. The exploration of Multimodal Interaction Systems within the realm of SEGs demonstrates their potential to revolutionize rehabilitation strategies, and their capacity to offer considerable advantages in terms of safety and sustained benefits for individuals undergoing therapy [15]. Using a proper software architecture allows better and faster development and allows new devices and reasonings to be easily added to the software.

Therefore, this work focuses on the following general research question: “How to create an SEG compliant with the needs of rehabilitation with various interaction devices?”

The objective of this paper is to introduce a multimodal software architecture for SEGs, called “123-SGR Architecture”, where the number “1” represents a single solution for multimodal interactive software architecture; the number “2” emphasizes a dual directionality of the interaction flow and two distinct flows of information (conscious/voluntary and unconscious/involuntary); and the number “3” highlights achieving all three functionalities (flexibility/equivalence, complementarity, and monitoring). “SGR” stands for Serious Game for Rehabilitation. The primary focus of this architecture is to effectively address the specific needs of therapists and patients in the domain of physical rehabilitation. Additionally, a proof of concept will be provided through its application in respiratory rehabilitation (RR) therapy. By implementing the proposed approach within the context of RR therapy, the aim is to demonstrate the practicality and effectiveness of the multimodal SEG architecture, while also validating its applicability and advantages in real-world rehabilitation settings.

Section 2 describes the 123-SGR architecture project. Then, Section 3 describes the application of 123-SGR in an SEG called I Blue It. Section 4 presented the assessment result with I Blue It. Then, the discussion is in Section 5. Finally, Section 6 described the conclusion and contribution of this work.

## 2. Materials and Methods

This section presents the 123-SGR architecture, a pivotal development in the context of Serious Games for Rehabilitation (SGRs). It encompasses essential features that play a significant role in enhancing SEGs for rehabilitation purposes.

The motivation behind focusing on specific aspects of the architecture stems from the objective of creating a robust and versatile framework for SEGs in rehabilitation. The incorporation of Decision, Action, Perception, and Interpretation states [18] ensures a comprehensive understanding of patient interactions, enabling personalized and adaptive therapy exercises. Additionally, the inclusion of a data structuring and storage module [19] is crucial for effectively processing and analyzing the diverse data generated via numerous modalities in rehabilitation settings.

The architecture prioritizes patient engagement and motivation by intercepting and adapting game controller signals based on physiological inputs [20]. Following the flow principle, this approach aims to maintain patient motivation throughout treatment. Moreover, the architecture enables player progress to be monitored and dynamic adjustments to be made to game difficulty levels [9,21], fostering a personalized and challenging gaming experience.

By incorporating fusion, fission, flexibility/equivalence, and complementarity functionalities [18], the architecture seamlessly integrates and coordinates various modalities, enhancing the overall effectiveness of rehabilitation interventions. Dynamic modality management at runtime [22] facilitates the adaptability and customization of gameplay to meet changing patient needs.

Leveraging elements from game design, such as mechanics, dynamics, and aesthetics [23], enriches the gaming experience, making it engaging and enjoyable for patients. The architecture ensures patient safety by dynamically adjusting game difficulty levels based on physiological data, such as heart rate [24]. The adaptation of game elements, including mechanics, level design, and parameters, based on patient responses [21], further enhances the individualized and therapeutic nature of Serious Exergames. Overall, the comprehensive integration of these functionalities addresses therapists’ and patients’ specific needs in physical rehabilitation, providing a compelling rationale for the design and implementation of the 123-SGR architecture.

**Figure 1:**
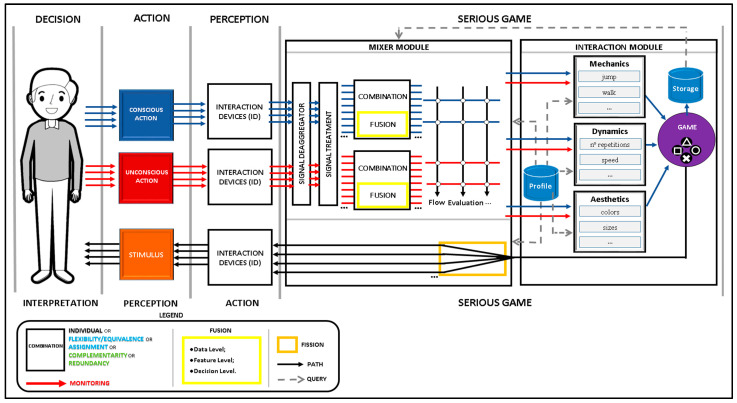
The 123-SGR architecture.

The 123-SGR architecture for multimodal SEGs encompasses three interaction flows: conscious flow, unconscious flow, and feedback flow (see Figure 1).

### 2.1. Conscious Flow

Conscious flow is what characterizes the purposeful manipulation of the modalities, performed by the player to generate a result in the exergame. Conscious flow goes to the following states:

Decision: The player consciously creates a message.

Action: The player performs the action that represents the thought message.

Perception: Messages are perceived by one, or multiple devices.

Serious Game: The multimodal SEG interprets the data and their meaning by using the Mixer and Interaction modules:

- Mixer Module: The signal management module consists of five cores: Signal Deaggregator, Signal Treatment, Combination, Adaptation Grid, and fission (the latter used only in the feedback flow):
  1. Signal Deaggregator: this core separates information so that it can be used in the game.
  2. Signal Treatment: this determines how the signal is collected (data sampling, filtering, etc.), whether there is a need for signal amplification via software, how valid data are extracted, and how these valid data are used in the game.
  3. Combination: This receives the signals from the devices and determines how each of them will proceed: (i) if flexible (one signal OR the other); (ii) with different assignments (one makes A, the other makes B); (iii) as a fusion of redundant modalities (one and one); (iv) as a fusion of complementary modalities (one and the other); (v) or directly (when there is only one signal). At the end of the combination of the signals, they are sent to the Adaptation Grid core.
  4. Adaptation Grid: this is responsible for performing the tests, parameterized by a therapist, to generate adaptations that can affect the mechanics, dynamics, and aesthetics of the SEG. It deals only with adaptations for flow purposes (maintaining player interest) and for physical evaluation (monitoring), but this core allows the addition of as many tests and adaptations as necessary, accounting for possible conflicts among triggers (values set by therapists that require changes to be made).
  5. Fission: this analyzes the feedback message from the core game and delivers it to the player via the devices available, for example, a monitor and a speaker sound box.
- Interaction Module: This is responsible for applying the adaptations and the interaction into the SEG. This module has 6 distinct cores:
  1. Mechanics: this is responsible for the actions to be performed, such as jumping or walking, based on the information received from the Adaptation Grid.
  2. Dynamics: this is responsible for the difficulty of the game; it controls parameters such as speed, number of repetitions, size of obstacle collision areas, height of target collision areas, score formula, etc.
  3. Aesthetics: this determines the visual aspects of the game, such as colors, shapes, and sizes of objects.
  4. Game: where the mechanical, dynamic, and aesthetic aspects are applied.
  5. Storage: this is where game data as well as device signals are recorded, separated by player and timeline.
  6. Profile: this is a core that serves the Adaptation Grid and the mechanics, dynamics, and aesthetics cores, so that the adaptations are made in accordance with users’ capabilities and pathologies.

### 2.2. Unconscious Flow

Unconscious flow captures the patient’s unconscious or unintentional physiological characteristics (sometimes beyond the desirable limit). For example, changes in heart rate and oxygen saturation. This interaction flow allows functionality to be monitored and is described through the states below.

Decision: in unconscious flow, there is no decision being made on purpose, but a reaction provoked by the conscious actions of the first flow; for this reason, the state “Decision” is not evidenced in this flow.

Action: the player’s physiological state is captured by the system regardless of the player’s interactions.

Perception: the same as the conscious flow behavior; however, signals (messages) are captured by other specific devices, or other signals from existing devices are reasoned with.

Serious Game: at this state, the unconscious flow goes through the same modules with the same functionalities as the conscious flow, but they are concerned with unintentional signals for safety monitoring goals.

### 2.3. Feedback Flow

Upon receiving signals from the conscious and unconscious flows, the necessary modifications to the game are applied, and to complete the interaction cycle, the game returns a response, showing the patient what changed in the game. The feedback flow is described below.

Serious Game: This is responsible for analyzing game occurrences and producing feedback to the player:

- Game: this core sends a response message to the Mixer module.
- Profile: this is a core that helps the Mixer module to send messages consistent with the player’s profile. For instance, if the player has a hearing impairment condition the Mixer module should not send a response on an audio channel or send it at a specific audible frequency.
- Mixer Module: this now triggers the fifth core, fission, which analyzes the possibilities and selects the devices to deliver the game response.

Action: the fissioned response is then passed to available devices.

Perception: the player’s senses capture the response.

Interpretation: the player interprets what the response given by the game meant and the multimodal interaction cycle repeats until the end of the game execution.

The definitions of the Mixer and Interaction modules assume a crucial role within the 123-SGR architecture. The Mixer module consists of five cores and is responsible for signal management, combination, and adaptation processes, ensuring an efficient integration of diverse modalities to enhance gameplay. Conversely, the Interaction module, with its six distinct cores, governs game mechanics, dynamics, aesthetics, and interactions, resulting in a comprehensive and personalized gaming experience. These modules collaboratively enable seamless interaction between players and the SEG, thereby empowering effective rehabilitation interventions and monitoring functionalities throughout the conscious, unconscious, and feedback flows.

### 2.4. Features of the 123-SGR Architecture

The 123-SGR can be used in various areas of physical rehabilitation, particularly in respiratory rehabilitation. Processing multimodal inputs can be performed at various levels of the information:

- At the signal level, where the voltage and current in analogic or digital forms are manipulated.
- At the data level, where the signal level is made available as a sequence of values in the computer.
- At the feature level, where elements of game design that refer to parameters such as target height, size of obstacles, space between objects, and game speed, for instance are manipulated.
- At the decision level, where the signals from patient interaction are applied to modifications in the game.

123-SGR architecture works at the data level upwards. The following existing game and architectural requirements were incorporated (Table 1):

### 2.5. Quality Attribute Scenarios

This section explores quality attribute scenarios [26]. Quality attribute scenarios objectively assess the quality requirements of a proposed architecture, thereby ensuring its effectiveness and suitability for the domain, in this case physical rehabilitation.

#### 2.5.1. Availability Scenario

Quality attribute scenarios measure the architecture’s effectiveness. By creating an immersive and engaging gaming experience through rigorous evaluation, patients are motivated to actively participate in their rehabilitation journey. By quantifying the impact of these design elements on user engagement, optimization of the architecture to achieve the desired therapeutic outcomes is facilitated.

Justification: The therapy is important for patients’ recovery. System disruptions can hinder this progress.

Success Criteria:

1. Uptime: “I Blue It” targets a minimum 95.0% uptime.
2. Rapid Recovery: system recovery is achieved within minutes.
3. Proactive Notifications: users are alerted about potential disruptions.
4. Redundancy: key components, like devices and databases, have redundancy.

Strategies:

1. Continuously monitor for availability.
2. Use the “123-SGR” architecture for resilience and adaptability.
3. Set clear maintenance protocols.

Example of Quality Attribute Scenario for Availability:

- Source: an external device.
- Stimulus: a failure (crash).
- Artifact: communication channels.
- Environment: normal operation.
- Response:
  ○ Log the failure.
  ○ Notify relevant entities.
  ○ Address the failure and mitigate.
- Response measure:
  ○ The failure is addressed within minutes.

#### 2.5.2. Accuracy Scenario

The Decision, Action, Perception, and Interpretation states contribute to the accuracy and responsiveness.

Definition: “I Blue It” processes user inputs accurately.

Justification: precise processing is key for user satisfaction.

Success Criteria:

1. Input Processing: system accurately interprets inputs.
2. Objective Metrics: system accuracy is quantifiable.
3. Alignment: system accuracy aligns with user expectations.

Strategies:

1. Emphasize algorithm accuracy.
2. Use quality attribute scenarios.
3. Adjust based on feedback.

Example of an Accuracy Scenario:

- Source: patient interacting with the system.
- Stimulus: user input during a session.
- Artifact: “I Blue It” SEG.
- Environment: active user interaction session.
- Response:
  ○ Processes input using Decision, Action, Perception, and Interpretation.
  ○ Provide accurate feedback.
- Response Measure:
  ○ Process inputs with 95% accuracy.

#### 2.5.3. Adaptation and Flexibility Scenario

Quality attribute scenarios assess the architecture’s adaptability to devices like PITACO, MANO-BD, and extensor belts. Adaptability ensures tailored user experiences and signal integration from diverse modalities.

Definition: “I Blue It” adapts to various interaction modalities.

Justification: adaptation enhances personalized rehabilitation.

Success Criteria:

1. Modal Adaptability: the system transitions between modalities.
2. User Experience: seamless interactions.
3. Signal Integration: modalities are integrated for comprehensive data.

Strategies:

1. Recognize and adapt to different devices.
2. Integrate diverse signals.
3. Optimize user experience across different devices.

Example of an Adaptation and Flexibility Scenario:

- Source: patient using a specific device, e.g., PITACO.
- Stimulus: device interaction.
- Artifact: “I Blue It” SEG.
- Environment: rehabilitation session.
- Response:
  ○ The system detects and adapts to the device.
  ○ Integrates device signals.
  ○ Provides tailored interaction.
- Response Measure:
  ○ System adapts according to measures detected by the devices.

#### 2.5.4. Interoperability Scenario

Monitoring of accurate physiological data, like heart rate, is vital during rehabilitation sessions. Quality attribute scenarios ensure data precision and reliability.

Definition: “I Blue It” monitors physiological data accurately.

Justification: accurate monitoring, such as heart rate tracking, is essential for patient safety.

Success Criteria:

1. Data Accuracy: Precise physiological measurements are provided.
2. Real-Time Monitoring: game features adapt according to physiological data.
3. Reliability: data acquisition and processing are consistent.
4. Patient Safety: patient well-being is prioritized.

Strategies:

1. Validate data for accuracy.
2. Monitor and adjust based on safety.

Example of Interoperability Scenario:

- Source: physiological sensors worn by the patient.
- Stimulus: data capture, e.g., heart rate.
- Artifact: Mixer module of the game’s architecture.
- Environment: rehabilitation session.
- Response:
  ○ Capture and process data.
  ○ Therapists monitor.
- Response Measure:
  ○ Game parameters change immediately after reasoning.

Incorporating quality attribute scenarios strengthens the architecture’s foundation, ensuring its applicability in real-world rehabilitation settings. These scenarios validate the architecture’s patient-centric, adaptive, and engaging nature.

## 3. Implementation Results: A New Version of an SEG

The 123-SGR architecture was utilized in the adaptation of the Serious Exergame (SEG) titled “I Blue It” [12]. This SEG primarily operated within conscious flow, where the only control involved the use of an interaction device (ID) named PITACO, responsible for capturing the player’s respiratory airflow using a pneumotachograph. As “I Blue It” is open-source [27,28], it provides accessibility for accessing and modifying its code to align with the 123-SGR architecture, further enhancing its adaptability and potential for widespread use in rehabilitation settings.

### 3.1. SEG I Blue It

**Figure 2:**
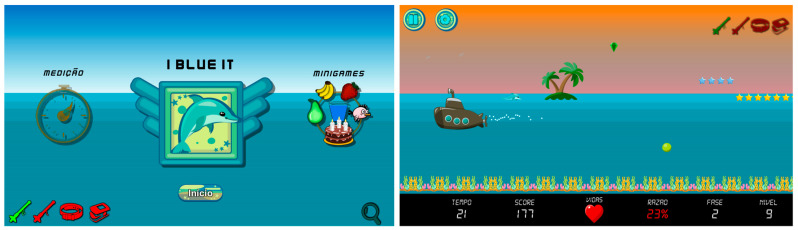
I Blue It 4.5.

The “I Blue It” respiratory rehabilitation SEG (see Figure 2) is designed to support individuals with respiratory illnesses throughout their rehabilitation journey. Players engage with the game as a dolphin character, called Blue, controlling its movements through specific breathing techniques: exhaling submerges Blue, while inhaling enables Blue to jump. Players navigate through different game levels, avoiding obstacles and collecting targets and bonuses. The previous version of I Blue It and its device is a good candidate to become a multimodal SEG because:

1. It is an open-source project freely available in C# and is Unity^®^ engine-based.
2. The assembly of the ID is also available and detached freely.
3. It benefits greatly from the ability to use other IDs because not all respiratory dysfunctions deal with airflow, but also with air pressure and other aspects. Thus, it benefits from multimodal flexibility.
4. It benefits greatly from the ability to use other IDs because respiratory exercises can make someone faint; this can be identified through peripheral oxygen saturation or heart rate, for instance. Thus, it benefits from multimodal monitoring.
5. It benefits greatly from the ability to use IDs alongside others because some exercises aim to change patients’ breathing, and not only the amount of airflow one can produce but also how muscles, postures, and other measures are adjusted. Thus, it benefits from multimodal complementarity.

The latest version of I Blue It complies with the 123-SGR architecture, incorporating various interaction devices such as the Pneumotachograph Device (PITACO), Manovacuometer Device (MANO-BD), Extensor Belt Device, and Pulse Oximeter Device. These devices measure and monitor essential respiratory parameters, encouraging specific respiratory actions, including expiratory flow, inspiratory flow, and maintaining a steady flow, all of which are crucial for effective respiratory rehabilitation.

### 3.2. Interaction Devices (ID)

**Figure 3:**
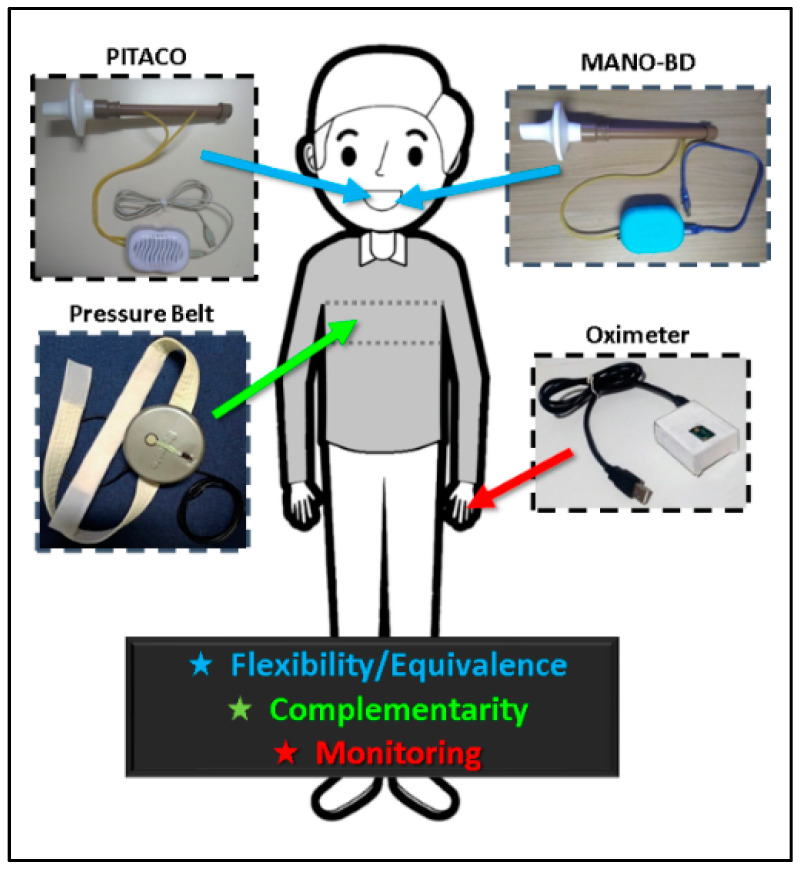
Positioning the interaction devices (ID) on the player.

To achieve all required functionalities, we needed a variety of multimodal IDs useful for respiratory rehabilitation:

- Using the PITACO construction manual [12] as a basis, it was possible to reproduce it (Figure 3).
- Based on the PITACO ID, we created the MANO-BD (Digital Bidirectional Manovacuometer), an ID that captures the air pressure blown into the device. A MANO-BD ID is composed of an absolute pressure sensor, model MPX5700; an Arduino for electronic prototyping, model Uno Revision 3; a connection between the sensor and Arduino, which is made using flat cables; a PVC tube into which the player blows; and a PVC cap to prevent the air from escaping.
- A Pressure Belt ID was created to measure the pressure exerted by the chest or abdomen of the player against the belt while using the game. The Pressure Belt is composed of a resistive force sensor, model FSR402; an Arduino, model Uno Revision 3; and a connection between the sensor and Arduino, which is made using flat cables located inside a rigid metal can. The Pressure Belt uses an adjustable nylon strap with Velcro around the chest holding the ID in place to measure respiratory effort and respiratory rate.
- An Oximeter ID was built to measure the blood oxygen saturation and the heart rate of the player while using the game. The Oximeter ID is composed of an oximetry sensor, model MAX30102; a connection bar to weld the sensor; an Arduino, Model Nano Revision 3; and a connection between the sensor and Arduino, which is made with flat cables on a 70-point mini protoboard.

It must be noted that the validation of the IDs with commercially available medical instruments is beyond the scope of this paper. Figure 3 exemplifies the positioning of the selected ID on the player, which the I Blue It SEG uses in its multimodal version thanks to 123-SGR.

### 3.3. Multimodality Analysis

ID for conscious actions can be seen as providers of flexibility/equivalence or complementarity functionalities, and IDs for unconscious actions can be seen as providers of monitoring functionality, as they can indirectly show indications of tiredness and dizziness. IDs were analyzed to see which ones can provide specific actions that can be used in gameplay (see Table 2).

By analyzing Table 2, it can be seen that most IDs listed are equivalent to most conscious actions, but PITACO and MANO-BD cannot be used together because they use the same pathway to capture respiratory flow (the mouth).

Some of the many possible modality combinations to generate complementarity are shown in Table 3.

- Combination 01: can estimate whether a player is commanding the game through predominantly thoracic or predominantly diaphragmatic breathing and determines the volume of the player’s respiratory flow.
- Combination 02: this can also estimate whether a player is commanding the game through predominantly thoracic breathing, or diaphragmatic, and provides the measure of the pressure exerted by the musculature of the player’s respiratory tract.

### 3.4. Conscious Flow

The player thinks about and performs a breathing maneuver to control the dolphin, Blue, and this maneuver is perceived by one ID or a combination of IDs (e.g., PITACO or MANO-BD or PITACO + Pressure Belt, etc.), as shown in Table 2, previously selected by the therapist considering the availability of IDs and the therapy goals.

### 3.5. Unconscious Flow

The following describes the analysis regarding the unconscious flow for the multimodal I Blue It SEG:

Decision: there is no conscious decision being made by the player in this flow.

Action: the player’s blood oxygen saturation can vary depending on the respiratory disease or breathing pattern.

Perception: oxygen saturation in the blood is perceived by the Oximeter ID.

**Figure 4:**
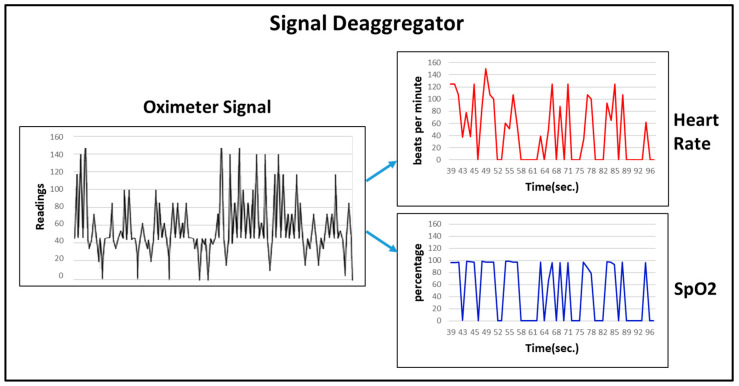
Signal Deaggregator—Oximeter.

Multimodal I Blue It: interpretation is performed through the components below:

- Mixer Module:
  1. Signal Deaggregator: used in the Oximeter, whose signal is composed of the player’s arterial oxygen saturation (SpO2) and the heart rate (HR) (see Figure 4).
  2. Signal Treatment: Amplification: not required. Sampling: 98/min. In-game use: speed parameter (default value: 10/min).
  3. Adaptation Grid: The SEG adapts according to the percentage of the player’s SpO2. If it falls in a given range, the speed of the game is decreased; if it falls below a second safety threshold, the game is interrupted to avoid implications to the player’s health.
- Interaction Module
  1. Mechanics: responsible for changing the speed of the game.
  2. Dynamics: generates an easier level or an interruption.
  3. Aesthetics: not used.
  4. Game: multimodal I Blue It receives and applies the elements of the previous three cores.
  5. Storage: game data and ID signals are all stored in a player-separated record chronologically.

### 3.6. Feedback Flow

The feedback flow is explained below:

Game: sends a reply message to the Mixer module’s fission core.

- Mixer Module: this now triggers the fifth core, fission, which combines the message returned by the game and the player profile, and delivers it to the player through the available and appropriate devices, such as a monitor and a sound box.

Action: the computed response is then passed to the selected devices.

Perception: the player’s senses capture the response.

Interpretation: the player interprets the response given by the game and the interaction cycle repeats until the end of the game execution.

### 3.7. Combination Processes in the Multimodal I Blue It SEG

Within the Mixer module implemented in the multimodal I Blue It, there is the Combination core. As soon as the signals enter this core, several forms of automatic combination are possible: individual, flexibility/equivalence, assignment, complementarity, and redundancy.

Individual: if only one signal arrives (for example, PITACO’s), a combination does not occur and the signal exits the same way it entered.

Flexibility: if two or more equivalent signals arrive, a combination may occur that allows only one of the signals to go forward.

Assignment: With two or more non-equivalent signals (e.g., PITACO’s and the Pressure Belt’s), the signal of each of them can be used for different purposes/assignments, i.e., signals come and go independently of the core, each one with a different purpose. Another example of an assignment is when a single ID has more than one signal, such as the Oximeter that generates a heart rate signal (HR) and a blood oxygen saturation (SpO2) signal. The two signals enter the Combination core and the two come out, each with a different assignment.

Complementarity: If two or more non-equivalent signals arrive at the core, besides being able to record both of them to correlate one to another, a merge may occur that generates only one output signal as a result. As an example, the combination of the PITACO ID and the Pressure Belt ID can be parameterized to occur in the following ways (only one of these forms of fusion is usable at a time, previously chosen in the parameter file):

1. The association of the signals of both is performed, and the resulting signal is the junction of 75% of PITACO’s and 25% of the Pressure Belt’s signals, making PITACO the preferred ID (percentages are customizable parameters, the values entered here are for example only).
2. The association of the signals of both is performed, and the resulting signal is the junction of 25% of PITACO’s and 75% of the Pressure Belt’s signals, making the belt the preferred ID (values just for example).
3. The negative values of PITACO and the positive values of the Pressure Belt are used.
4. The positive values of PITACO and the negative values of the Pressure Belt are used.

**Figure 5:**
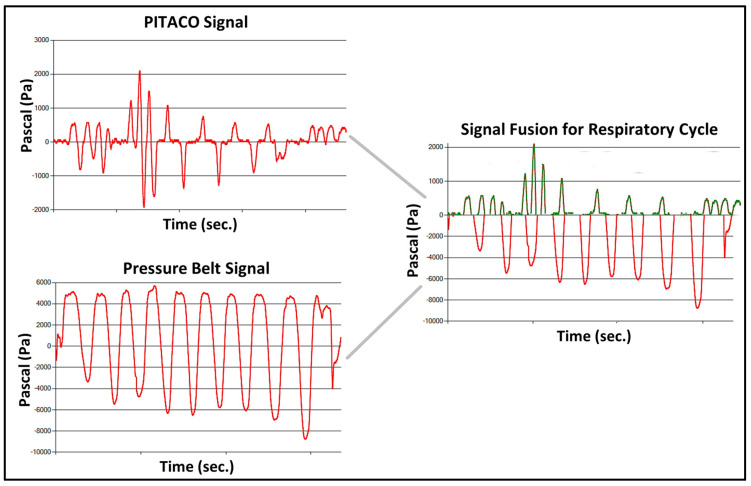
Complementarity—using different parts of each signal.

Figure 5 shows option 3 above, where each ID has signals complementary to the other; this merge may be useful when one is better at identifying the nuances of an inspiratory maneuver, while another ID may be better at measuring an expiratory maneuver, and it is necessary to use the signals from each ID for one part of the respiratory cycle (different parts/functions of the same task).

Redundancy: Finally, if two or more “equal” signals reach the core, a fusion may occur, generating only one resulting signal (a combination commonly used to correct faulty, intermittent signals). The only type of multimodal combination “with signal fusion” applied in multimodal I Blue It is complementarity. The possibility of merging for data redundancy has not been considered.

Fission Process for the Multimodal I Blue It SEG: This feedback is triggered via game mechanics as a message and the core divides the message consistently into each type of feedback requested and sends each fragment to appropriate devices. For example, if the message is “The target has been hit”.

Visual feedback: the image of the collected target is sent to a visual output device, such as a monitor.

Sound feedback: the collected target sound is sent to a sound output device, such as a loudspeaker.

## 4. Assessment Result: Is the New Multimodal SEG Any Better?

To assess the utility of all multimodal functionalities and of the 123-SGR architecture, a questionnaire was sent to 399 professionals who have a health background (in this case, respiratory and/or motor rehabilitation, physical educators, speech therapists, etc.) or technical background (in this case, engineering or game design).

Twenty-seven (27) professionals answered the questionnaire (6.77%), which had questions regarding the importance, necessity, and practical criteria on a five (5)-point Likert Scale. The questionnaire had five demographic questions; three (3) questions regarding the importance, necessity, and practical issues of the multimodal concept; three (3) questions for each functionality regarding some criteria (necessity, importance, and practical issues); and two (2) open questions on the positives/advantages and negatives/disadvantages of such multimodal approach. Table 4 presents the statistics for the most common answer (mode), the average score (mean), and the deviation for each criterion of each modality feature.

The questionnaire was applied remotely (online) and presented nine demonstrative videos to illustrate the IDs and functionalities. The best mean value results for each criterion are highlighted in green and the overall worst result is highlighted in red (the only one that fell below 4.00 on a 1 to 5 Likert scale). From Table 4, all scores indicate agreement with the utility of all criteria. Multimodal interaction (as a whole) obtained the highest score for importance and complementarity received the lowest score for practicality. The need for multimodal interaction achieved the highest consensus while the practicality of monitoring achieved the lowest consensus.

It can be seen from Table 4 that 11 of the 12 questions achieved a score above 4.15, which is well above the center of the scale (3, on a scale from 1 to 5). These results indicate that the multimodal concept as a whole for I Blue It and every single multimodal feature individually were considered of great importance, great necessity, and great practicality.

When dividing the objective responses among health (see Table 5) and technology professionals (see Table 6), the mean evaluations of both groups were above 3.6 (scale from 1 to 5), which means a positive agreement (above 3, the center of the scale). For health professionals, the most important functionality was flexibility/equivalence (in green in Table 5), which may be due to their vision of the various target audiences benefiting from different and equivalent forms of control/monitoring.

For health professionals, complementarity is the second most important and necessary feature, perhaps because it represents a general concept of interaction, whose applicability is not shown in practice but is crucial to the professionals in this area. On the other hand, professionals from the technology area considered the general concept of multimodal interaction as the most important feature, perhaps because it is closely linked to computer systems in general and that may have led these professionals to better understand their potential.

For health professionals, the complementarity feature is the least practical one, perhaps because it usually means a second device must be worn by the patient, which could be distracting, limiting, or cumbersome.

The least important and least necessary functionality for technology professionals is also complementarity, possibly because they do not see how important it is to correlate meaningful signals.

According to the mean scores, the professionals of the two groups reached a consensus when assessing that complementarity is the least-practical functionality since it requires synchronization among more than one ID, which can lead to difficulties in control and adaptation and extra costs, and present a hurdle to the player.

The greatest standard deviation among the responses of health professionals is related to the practicality of complementarity, possibly because it is subdivided into several areas, such as respiratory rehab, motor rehab, physical educators, speech therapists, etc. The greatest standard deviation among the responses of the professionals in the technology area is related to the practicality of monitoring, which may be due to a more accurate view of the sensor used in the Oximeter ID and its best reading condition, which requires the player’s finger to be strictly still during the game, which can represent difficulty in practice.

Dividing the objective responses among professionals “with” and “without” experience in respiratory rehabilitation (RR), all average evaluations were above 3.8, also suggesting a positive agreement on all functionalities. Experienced professionals consider the general concept of multimodal interaction to be the most important, that is, it is important in the application of RR techniques. For the professionals without experience in RR, the most important functionality is flexibility/equivalence, which may reflect that the explanation of functionality in the questionnaire was successful in demonstrating its importance to professionals outside the focus area of the multimodal I Blue It SG.

The least-important functionality for experienced professionals is flexibility/equivalence, and monitoring is the most important, which strengthens the need for players to take careful measures during rehabilitation. For professionals without experience in RR, the concept of complementarity is the least important and necessary, which may reflect the lack of experience in RR.

In the positive points/advantages question, 11 professionals highlighted monitoring as the most valuable point, which stresses the importance of this functionality and also stresses the need for a multimodal architecture such as 123-SGR. Practicality was also highlighted, even though this was a least-popular issue among the objective questions; this is a situation that exemplifies the disagreement between the professionals as shown by the standard deviation, which could be mitigated if the professionals had obtained the ID in person and did not evaluate it only through demonstrative videos.

In summary, there seems to be a consensus that all functionalities are important and necessary, but run into practical issues. There is also an agreement among the professionals on the importance and need for multimodal interactions within the scope of RR.

Among the answers to the open questions, one of the professionals noticed the absence of adaptations related to the monitoring of the player’s heart rate, a fact that was described in the questionnaire as information directed only to the therapist’s visualization.

As for the worry that the ID can allow air to escape through the mouth, both the PITACO and MANO-BD IDs use filters with proper inlets. The need for a nasal clip to prevent players from breathing through the nose can be a significant contribution, which can be directly incorporated by the therapists who may use the multimodal I Blue It SG.

It should be stressed that all IDs are prototype versions and, for this reason, the comfort, design, and proportions may not be the most fit for use. However, they are sufficient to confirm the potential of the architecture and as prototypes that will inspire improved IDs. Another point is that, as specified before, ID validation against medical instruments available in the market is out of the scope of this paper.

These comments were all related to the practicalities of using the SG, which proves that professionals can foresee using the multimodal SG in their practice.

## 5. Discussion

Multimodal SEGs for rehabilitation require a diversity of inputs that includes movements of limbs, speech, and breathing among others that are captured using unusual devices (other than a mouse and keyboard). Multimodal features such as flexibility/equivalence, and unconventional ways of controlling a game, particularly for some people with disabling pathologies, are the only possible methods of game/therapy access. This can therefore be seen as a limitation that Multimodal Interaction Systems help to solve.

### 5.1. About the 123-SGR Architecture

The 123-SGR architecture can also serve as a guide, a checklist of elements to be planned and developed. Each population is to be rehabilitated (stroke patients, elderly, Down syndrome, etc.), and each focus of the physical rehabilitation (motor, respiratory, voice, etc.) has its particular characteristics. The 123-SGR architecture allows the SEG to be adjustable because it leaves room for appropriate devices to be chosen, and for combinations of appropriate devices to be used in addition to all parameterization of the adaptation thresholds that are available for a therapist to customize.

The functionalities highlighted in the architecture are representations of CARE 15 (complementarity, assignment, redundancy, and equivalence) functionalities with a specific application in SEGs for physical rehabilitation. However, there are some features not described by the CARE functionalities but present in 123-SGR, such as:

- The flexibility functionality is seen not only as the equivalence functionality (CARE), which selects between two or more modalities. This functionality in 123-SGR also determines that the application can be flexible enough to lose or gain modalities at run time.
- The complementarity functionality not only allows the fusion of different modalities (as the CARE functionality describes), but determines that it can be produced in different ways, according to mathematical equations (+, −, *, /, potentiation, weighting, module, etc.) or by giving parts of the same task to each modality (as demonstrated by Figure 5).
- The functionalities of assignment and redundancy (CARE) can also be achieved via the 123-SGR architecture. However, they were not used in the SG for the area of physical rehabilitation, because the assignment (one performs task A, another performs task B) is usually implemented in software in general and does not allow a diversity of possibilities (since it does not perform fusion). A typical example of an assignment that occurs on multiple systems is use of a mouse device, typically used to click buttons/menus and a keyboard device to generate text in writing fields. Redundancy, on the other hand, has been interpreted as a CARE functionality to increase software reliability (higher availability) so that it merges but no information is supplemented. There is no extra information beyond that achieved with individual modalities/devices. For this reason, it did not gain relevance to the present SEG proof of concept.
- Monitoring is a functionality designed particularly for physical rehabilitation with SEGs because while requiring physical exercise, damage resulting from effort may occur that is not noticeable without the use of physiological sensors. Considered the single most necessary functionality according to the professionals (after the general concept of multimodal interaction), monitoring can be achieved through any of the four CARE functionalities: via equivalence, if choosing between one device (in this context, a device is a wrapper of one or more sensors) with the same sensory ability; via assignment, using more than one device, each with a different task; via redundancy, using equal devices with data united contingently (reliability); and via complementarity, in which different devices complement each other to achieve more complete information about the physiological state of the player.

All CARE functionalities were achieved using the 123-SGR architecture, both in the conscious and unconscious interaction flows.

### 5.2. About the Multimodal I Blue It SEG

The parameterization of optimal and acceptable oxygenation rates requires analysis by a therapist. On the other hand, it was noted that low oxygenation saturation always creates negative feedback for the player (pauses and on-screen alerts), while positive feedback could be implemented when rates are at good levels.

The merging of input modalities used in the multimodal I Blue It was performed only at the data level. Fusions could also be created at the characteristic level, such as to detect abdominal diaphragmatic breathing, through a complementary fusion of characteristics (such as from PITACO or MANO-BD) plus the Pressure Belt (coupled to the abdomen of the player), as shown in Table 2. Diaphragmatic abdominal breathing is one of the recognized practices to improve respiratory functions. The adaptation developed in the multimodal I Blue It to show the pressure exerted on the Pressure Belt is achieved by changing the character’s color to orange if the minimum percentage stipulated by the therapist is not reached. However, nothing prevents the player from expanding the abdomen without actually breathing in the requested way to achieve better gameplay and progression in the game. So, perhaps achieving abdominal diaphragmatic breathing through a fusion at the feature level could produce more satisfactory results.

In the multimodal I Blue It, the fission was managed only via the Unity^®^ engine, producing visual and audible feedback.

### 5.3. About the Evaluation

When answering the questionnaire, no controls were used that required the professionals (evaluators) to start the videos, nor to watch them to the end, which may have influenced the emergence of some doubts/comments already explained in the video. Thus, it would be of great help to use technologies that allow this control. As a counterpoint, the use of remote questionaries allowed us to reach more professionals than in a face-to-face evaluation.

In general, the data show that the I Blue It SEG and its new functionalities (including monitoring) have been highly appreciated by professionals from all areas. This finding indicates that multimodal SEGs for physical rehabilitation should be further explored and, for this, an architecture such as 123-SGR can facilitate the design and development of new SEGs.

The present I Blue It SEG was evaluated by professionals due to the need to know if such functionalities could somehow improve therapy (by providing extra information, flexibility, and safety). The data show a positive response on all accounts. However, patients’ views and feelings must also be evaluated.

## 6. Conclusions

Traditional physical rehabilitation can become tiring and monotonous. Serious Exergames (SEGs) can help maintain a patient’s interest and achieve effectiveness in treatment. However, current SEGs have not given due attention to three functionalities seen as important for therapists and patients: flexibility/equivalence (more than one control option); complementarity (combining information to be more clear about how rehabilitation exercises are being performed); and monitoring (capture of patient’s physiological data during the game so that the patient does not suffer any side effects from the actions exerted). To achieve these three functionalities, it was observed that several devices need to be worn by the patient.

Multimodal Interactive Systems have been dealing with a variety of ways to convey intentional commands to the system, but for SEGs we have demonstrated that intentional as well as unintentional signals are important, necessary, and practical, particularly when applied to Serious Games for Respiratory Rehabilitation (SGR).

Architectures were found for the construction of SEGs and Multimodal Interaction Systems; however, none of them aimed to build an SEG with all three mentioned functionalities. A new software architecture was created, the 123-SGR architecture, which allows the creation/adaptation of SEGs with a focus on physical rehabilitation so that they become multimodal SEGs and achieve all functionalities.

The 123-SGR architecture was used to adapt an existing SEG towards a multimodal one. Software elements were added and adapted, and new interaction devices (ID) were designed and built (MANO-BD, Pressure Belt, and Oximeter) for sensing the respiratory process, which provided multimodal interaction.

The multimodal SEG, I Blue It, was evaluated by health and technology professionals. We found that all scores were above the center of the scale and thus all evaluators positively agreed with all features; there are issues with game design and ID usability that must be taken care of; the multimodal I Blue It SEG has potential utility (importance, need, and practicality) in respiratory rehabilitation (RR); I Blue It was able to acquire the flexibility/equivalence, complementarity and monitoring functionalities; and; multimodalities are really important and necessary functionalities for a respiratory rehabilitation SEG.

The main contribution of this research is an architecture for multimodal SEG systems that is appropriate to the needs of the area of physical rehabilitation; is compliant with, but goes beyond, CARE functionalities; and that includes a flow to meet the monitoring functionality (for safety, for example). The 123-SGR architecture emphasizes flexible design aspects of Multimodal Interaction Systems, such as the various ways to combine input information (with selection functionalities and algebra) and these combinations are possible for both conscious and unconscious signals coming from the player. Further, the 123-SGR architecture also emphasizes the relationship of input information with the game elements (mechanics, dynamics, and aesthetics) of a digital SEG for rehabilitation.

Adopting the 123-SGR architecture will facilitate the development of new multimodal SEGs for physical rehabilitation, making them flexible, information-rich, and safe to use. Although tested on an SEG for respiratory rehabilitation, we argue that 123-SGR can be a useful architecture for all SEGs.

We expect that 123-SGR would require some coding for every new ID that is added as an input to the SEG. Other multimodal architectures present some high-level description language to describe and facilitate new devices being added, but we found that there is no standard or widely accepted description language for this, and they would also require some sort of coding. We also acknowledge that promoting the adaptations to just one SEG does not allow us to show all features and benefits of 123-SGR, but the chosen SEG was the one that we had free and easy access to.

Future work should consider handling all input signals and data to make the resulting SEG a more intelligent one. That is, the core module of 123-SGR architecture—the Mixer module—could incorporate Artificial Intelligence reasoning.

In addition, future research should investigate if all extra IDs could become a nuisance to the patient and compromise rehabilitation somehow. We suggest that unconscious flow should be made more evident to patients as a way to better conduct the treatment.

## Figures and Tables

**Table 1:** Architectural requirements and how and why they were achieved.

ID	Requirements	How	Why
R1	The objective was to read several sensors/devices (of the same type, or not) to connect and use them at the same time [8].	All devices are connected via the “Signal Deaggregator” module.	This is a requisite to achieve complementarity.
R2	The Decision, Action, Perception, and Interpretation states were used [18].	States represent groups of modules.	These are usual multimodal interaction processing states.
R3	A module for structuring and storing data has been incorporated [19].	A “Profile” module was included.	The “Profile” module is necessary to organize and store data efficiently, ensuring that relevant information is readily available for the application’s use.
R4	The SEG must intercept the signals from the game controllers before they are used in the game, simultaneously receive physiological signals from the player as inputs, then adapt the signals of the controllers accordingly to these physiological signs [20].	The “Mixer” module performs this task.	This interception process is crucial to ensure that the game responds appropriately to the player’s physiological state.
R5	Physiological data (e.g., heart rate) should be measured to reduce or increase the level of difficulty of the game, to promote patient safety [24].	All signals are dealt with through the same modules.	By measuring physiological data, the SEG can dynamically adjust the difficulty level of the game to match the patient’s current physical condition.
R6	An SEG should monitor the player’s progress over several matches and adapt the difficulty level according to the player’s current skill level (Flow) [9,21].	Progress monitoring is achieved through tracking the player’s performance in multiple matches and analyzing their skill level (Flow).	By monitoring the player’s progress, the SEG can dynamically adjust the game’s difficulty level, providing an engaging experience that challenges the player appropriately and encourages skill improvement.
R7	Fusion, fission, flexibility/equivalence, and complementarity must be allowed [18].	Specific modules to achieve this are in place.	It would allow any combination of the input signals.
R8	Signals should be mediated via a component that has the role of managing the paths of each modality [25].	It is achieved through grid evaluation.	It would allow the software design to decide what to do.
R9	Modalities should be allowed to be changed, removed, and added dynamically at runtime (gameplay) [22].	The “Signal Deaggregator” module allows a play and play feature.	It is a requirement for easy, practical, basic, daily use.
R10	The SEG should be loaded with predefined values. Parameters previously established by the therapist define the game controls and the settings of these controls based on the patient’s needs [22].	The “Profile” database stores all relevant data.	It is a requirement for easy, practical, basic, daily use.
R11	The four functionalities of the CARE [15] multimodal design have been incorporated in the form of flexibility/equivalence, complementarity, assignment, and redundancy functionalities.	See Section 5 for details.	To better achieve usability of multimodal systems.
R12	It is necessary to adapt elements of the game (mechanics, level design, parameters) so that they affect, for example, the interval between the appearance of virtual objects, or the size of these objects [21].	The architecture sought to show the three conceptual elements of game design through examples of what can be developed in each category, and this is a way to help divide and classify the parts of a game, such as mechanics, dynamics, and aesthetics.	Since it is an architecture for digital games, conceptual elements of game design were used [25]: mechanics: represents algorithms, rules, actions, and other game components; dynamics: resulting from the interaction between the player and mechanics; and aesthetics: represents what the game looks like, or even the subjective emotional response of the player while gaming. By dividing and classifying the parts, one understands better what is intended, which improves the design and development of the game/system.

**Table 2:** Relationship between interaction devices (ID) and conscious respiratory actions.

ID	Conscious Actions					
	Inspiration	Expiration	Flow Duration	Flow Volume	Strength	Pressure
PITACO	x	x	x	x	x	
MANO-BD	x	x	x		x	x
Pressure Belt	x	x	x			

**Table 3:** ID combinations towards complementarity.

Complementarity (and)					
ID	Inspiration	Expiration	Flow Duration	Flow Volume	Pressure
Combination 01					
PITACO	x	x	x	x	
Pressure Belt	x	x	x		
Combination 02					
MANO-BD	x	x	x		x
Pressure Belt	x	x	x		

**Table 4:** Statistical analysis of all professionals’ objective answers (*n* = 27).

Question			Mode [1–5]	Mean [1–5]	Standard Deviation
Q1	Multimodal Interaction	Important	5	**4.67**	0.55
Q2	Multimodal Interaction	Necessary	5	4.52	**0.51**
Q3	Multimodal Interaction	Practical	5	4.19	0.96
Q4	Flexibility/Equivalence	Important	5	**4.59**	0.84
Q5	Flexibility/Equivalence	Necessary	5	4.37	0.93
Q6	Flexibility/Equivalence	Practical	5	4.19	1.08
Q7	Complementarity	Important	5	**4.44**	1.01
Q8	Complementarity	Necessary	5	4.3	1.03
IQ9	Complementarity	Practical	5	**3.89**	1.19
Q10	Monitoring	Important	5	**4.52**	1.01
Q11	Monitoring	Necessary	5	4.48	1.01
Q12	Monitoring	Practical	5	4.15	**1.2**

**Table 5:** Statistical analysis of health professionals’ objective answers. (*n* = 13).

Health Professionals					
Question			Mode [1–5]	Mean [1–5]	Standard Deviation
Q1	Multimodal Interaction	Important	5	4.54	0.52
Q2	Multimodal Interaction	Necessary	4	4.46	0.52
Q3	Multimodal Interaction	Practical	5	4.31	0.75
Q4	Flexibility/Equivalence	Important	5	**4.77**	**0.44**
Q5	Flexibility/Equivalence	Necessary	4	4.46	0.52
Q6	Flexibility/Equivalence	Practical	5	4.46	0.66
Q7	Complementarity	Important	5	4.69	0.48
Q8	Complementarity	Necessary	5	4.62	0.51
Q9	Complementarity	Practical	5	**4.23**	**0.83**
Q10	Monitoring	Important	5	4.69	0.48
Q11	Monitoring	Necessary	5	4.69	0.48
Q12	Monitoring	Practical	5	4.54	0.66

**Table 6:** Statistical analysis of technology professionals’ objective answers. (*n* = 14).

Technology Professionals					
Question			Mode [1–5]	Mean [1–5]	Standard Deviation
Q1	Multimodal Interaction	Important	5	**4.77**	0.6
Q2	Multimodal Interaction	Necessary	5	4.54	**0.52**
Q3	Multimodal Interaction	Practical	5	4.23	1.01
Q4	Flexibility/Equivalence	Important	5	4.38	1.12
Q5	Flexibility/Equivalence	Necessary	5	4.23	1.24
Q6	Flexibility/Equivalence	Practical	5	4.08	1.26
Q7	Complementarity	Important	5	4.15	1.34
Q8	Complementarity	Necessary	5	3.92	1.32
Q9	Complementarity	Practical	5	**3.69**	1.38
Q10	Monitoring	Important	5	4.31	1.38
Q11	Monitoring	Necessary	5	4.23	1.36
Q12	Monitoring	Practical	5	3.92	**1.44**

## Data Availability

The data presented in this study is available on request from the corresponding author. The data is not publicly available due to ongoing future studies.
